# Supplementary material for: Chromothripsis during telomere crisis is independent of NHEJ, and consistent with a replicative origin
Source: Genome Res. 2019 May;29(5):737–49. doi: 10.1101/gr.240705.118 (PMC6499312; doi:10.1101/gr.240705.118)
Supplement: Supplemental Material [file supp_gr.240705.118_Supplemental_file_1.zip › contigs/annotated_contigs/DB51/contig.2.DB51_length_498_mean_cov_6.89759036145.docx]

**DB51_length_498_mean_cov_6.89759036145**

```
TGTTATGTGTGAATTTGATCCTGCCATTATGATTTTAGCTGGTTATTTTGCTCGTTAGTTTATGCAGTTTCTTCCTCACCTTGATGGTC
 >chr6:80058283-80058626 - E=6e-195
TTTACAATTTGGCATGTTTTTGCAGTGGCTGGTACCAGTTGTTCCTTTCCATGTTTAGTGCTTCCTTCAGGAGCTCTTGTAAGGCAGGC

CCAGTGGTGACAAAATCCCTCAGCATTTTCTTGTCTGTAAAGGATTTTATTTCTCCTTCACTTATGAAGCTTAGTTTGGCTGGGTATGA

AATTCTAGGTTGAAAATTCTTCTGTTTAAGAATGTTAAATATTGGCCCCCACTCTCTTCTGGCTTGTAGAGT|TTCT|TTTTGCTCAAA
                                                                         >chr6:12268997
ATTGCTTTGGCTATTTGGAGCCTTTTCTGGTTTCATATGAATTTTAGGATCAACTTTTTCTATTCCTGTGAAATATAATTTTGTGAAAA
-12269156 + E=4e-84
ATATCATTGGTATTTTGATAGGGATTGTATTGAATCTATAGATGACTTTGAGTAG
```
